# Applications of human-machine collaborative decision-making: A review of research with recent developments

**DOI:** 10.1016/j.isci.2026.116056

**Published:** 2026-05-22

**Authors:** Yun Luo, Yanpuze Hao, Haochen Gong, Bo Li

**Affiliations:** 1 School of Systems Engineering, National University of Defense Technology, Changsha 410073, China

**Keywords:** Applied sciences, Computer science, Computing methodology, Artificial intelligence, Social sciences, Decision science, Linguistics

## Abstract

This study comprehensively reviews human-machine collaborative decision-making (HMCD) methods and applications across management science, the military, healthcare, and manufacturing. We propose a dual-layer analytical framework. The first layer decomposes HMCD into four sequential stages, namely attribute determination, weight assignment, information aggregation, and decision-making. The second layer identifies four cross-cutting collaboration mechanisms, namely role configuration, interaction and deliberation, trust and explanation, and authority and responsibility migration. A feedback loop connects decision outcomes to earlier stages, capturing iterative adaptation between agents. Using this framework as an analytical lens, we synthesize methods and applications, examine stage-specific collaboration patterns across domains, and trace how failures propagate through mechanism dependencies. This review provides a structured map of HMCD research and guidance for collaborative system design.

## Graphical abstract

**Figure:**
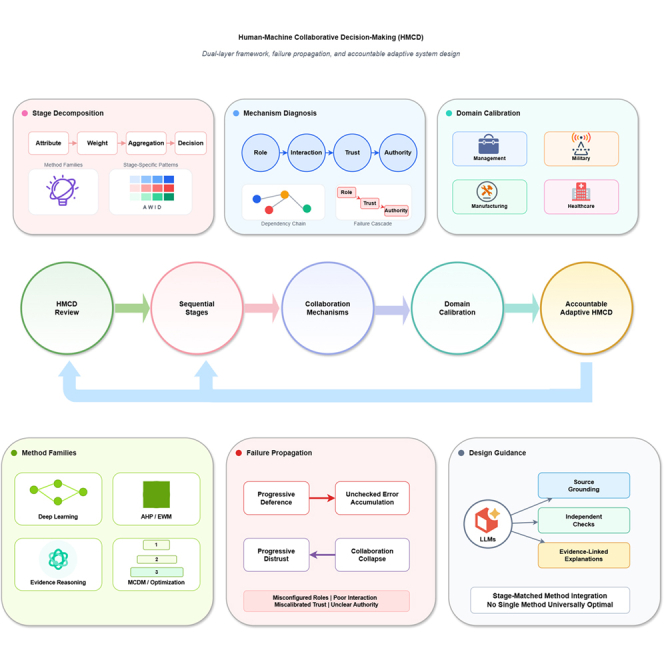

## Introduction

Artificial intelligence (AI) technologies are increasingly integrated into decision-making across industry, healthcare, management, and the military. Yet machine intelligence remains limited in contextual reasoning and ethical judgment, while human intelligence alone proves insufficient for complex, dynamic, and high-dimensional problems.^1^^,^^2^^,^^3^ Human-machine collaborative decision-making (HMCD) has emerged as a paradigm that combines human cognition with machine capabilities to address these complementary limitations.

The conceptual foundation of HMCD traces back to Licklider’s “man-computer symbiosis” in the 1960s^4^ and Qian’s framework for open complex giant systems,^5^ both envisioning coordinated participation between human and machine intelligence. While decision support systems have existed since the late 20th century, recent AI advancements have extended machine capabilities well beyond rule-based systems, enabling machines to assume central roles in complex decision environments,^6^^,^^7^ as validated across computer science, management, and information systems.^8^^,^^9^^,^^10^^,^^11^^,^^12^

We define HMCD as a process in which human contextual judgment and AI analytical capabilities are integrated through bidirectional exchange and adaptive coordination. This distinguishes collaboration from mere assistance, where machine outputs are accepted or rejected without mutual adjustment.^13^^,^^14^ The HMCD process unfolds through multiple stages, each requiring alignment between human roles, task contexts, and decision goals.

The theoretical foundations of HMCD draw on Simon’s stage-based decision theory^15^ and multi-criteria decision-making (MCDM) theory,^16^^,^^17^ which together provide a process view through criteria definition, weight determination, preference aggregation, and alternative selection. Fitts’ functional allocation framework distinguishes tasks suited to humans from those suited to machines.^18^ These perspectives are necessary but not sufficient, because HMCD also involves semantic alignment and authority separation between agents with fundamentally different cognitive encoding, problems that do not arise when participants share common representational systems. Addressing these problems requires distributed cognition,^19^^,^^20^ trust calibration,^21^^,^^22^ team cognition,^23^ and co-adaptation^24^ perspectives, which are developed in the theoretical grounding section.

Existing reviews typically organize HMCD studies by domain or list methods descriptively, leaving open at which stage collaboration matters most, what mechanisms determine success or failure, and how decision authority should shift as situations evolve. Recent developments further challenge existing approaches. Large language models (LLMs) turn human-machine interaction into conversational deliberation while introducing hallucination risks.^25^^,^^26^ Value alignment methods support the reconciliation of human preferences with machine optimization.^27^^,^^28^ Embodied intelligence deepens human-machine coupling in physical decision environments.^29^^,^^30^

**Figure 1:**
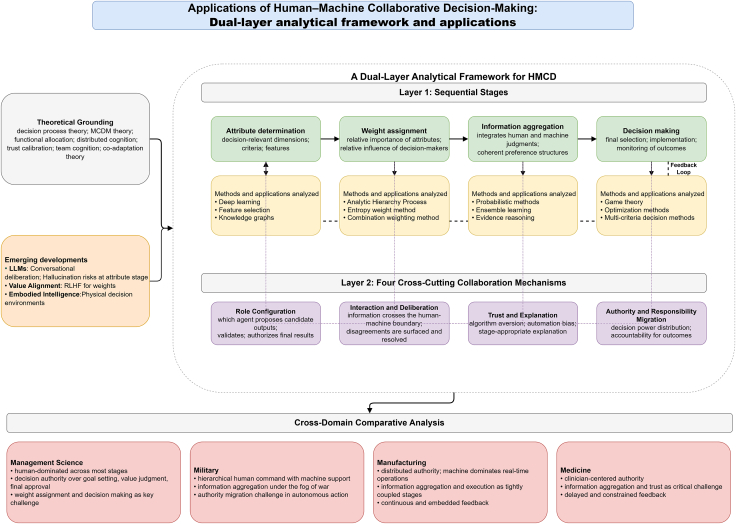
Dual-layer analytical framework for human-machine collaborative decision-making The framework integrates two analytical layers with a cross-domain comparative panel. Layer 1 decomposes HMCD into four sequential stages, namely attribute determination, weight assignment, information aggregation, and decision-making, each associated with representative methods examined in this review. A feedback loop links decision outcomes back to upstream stages to enable iterative adaptation. Layer 2 identifies four cross-cutting collaboration mechanisms, namely role configuration, interaction and deliberation, trust and explanation, and authority and responsibility migration, which operate within and across all stages. The lower image maps the framework onto management science, the military, manufacturing, and medicine, summarizing dominant role configurations and the most pressing collaboration challenge in each domain.

To address these gaps, we propose a dual-layer analytical framework. The first layer decomposes HMCD into four sequential stages, namely attribute determination, weight assignment, information aggregation, and decision-making. The second layer identifies four collaboration mechanisms that operate across stages, namely role configuration, interaction and deliberation, trust and explanation, and authority and responsibility migration. A feedback loop connects outcomes to earlier stages, enabling error correction and mutual adaptation. Figure 1 provides an overview of this dual-layer framework and its application across the four domains examined in this review.

## Research scope

We conducted a comprehensive narrative review of HMCD literature. Studies were retrieved from peer-reviewed journals indexed in the Web of Science database with coverage up to early 2026, using keyword combinations covering HMCD methodologies and their application contexts. Selection emphasized conceptual representativeness and methodological diversity rather than exhaustive coverage.

We focused on four domains with significant HMCD research activity, namely management science, the military, healthcare, and manufacturing. These domains were selected based on publication frequency, methodological maturity, and diversity of decision-making contexts. Other domains such as education, agriculture, and transportation were excluded due to sparse literature and the absence of established HMCD frameworks. Both qualitative and quantitative studies were included.

Studies were screened based on titles and abstracts, with inclusion criteria requiring explicit involvement of both human and machine agents in a collaborative decision-making process. Selected studies were categorized by domain and further analyzed according to the dual-layer framework introduced in the “a dual-layer analytical framework for HMCD” section to enable cross-domain and cross-stage comparison.

## A dual-layer analytical framework for HMCD

### Theoretical grounding

This study establishes a dual-layer analytical structure for HMCD. The first layer decomposes the decision process into sequential stages. The second layer identifies collaboration mechanisms that operate within and across these stages. The following subsections develop each layer and explain why both are necessary.

#### Stage decomposition and its limitations

Building on Simon’s decision model and MCDM theory,^15^^,^^16^ we identify four core stages. Attribute determination specifies decision-relevant dimensions. Weight assignment determines the relative importance of attributes. Information aggregation integrates human and machine judgments. Decision-making produces final choices and implements them.

Stage decomposition identifies where collaboration problems occur but not how they operate. Because human and machine agents encode knowledge through fundamentally different systems, vectors and probability distributions on one side, semantic categories and causal narratives on the other, each stage requires negotiation over what information means, how reliable the outputs are, and who holds final confirmation power. A second analytical layer of cross-cutting collaboration mechanisms is therefore needed.

#### Four cross-cutting collaboration mechanisms

We identify four mechanisms that operate across all stages, each addressing a distinct question about human-machine coordination.

(1) Role configuration: Fitts’ framework allocates tasks statically between humans and machines^18^ but does not address how roles should change across stages or how they should be renegotiated as capabilities evolve. Role configuration extends this allocation into a dynamic dimension. At each stage, it examines which agent proposes candidate outputs, which validates them, which authorizes final results, and under what conditions assignments should be adjusted.
(2) Interaction and deliberation: distributed cognition theory holds that collaboration effectiveness depends on information transmission quality across agent boundaries rather than on individual capabilities.^31^ The representational gap identified in stage decomposition and its limitations creates concrete translation problems at every stage. Machine-discovered features may lack interpretable labels, human preferences may resist formalization, and probabilistic uncertainty may not map onto human risk intuitions. The interaction mechanism examines how information crosses the human-machine boundary, how machine outputs are transformed into comprehensible forms, how disagreements are resolved, and where information loss leads to collaboration failure.
(3) Trust and explanation: trust mediates human willingness to rely on machine outputs. Algorithm aversion leads users to undervalue accurate recommendations,^32^ while automation bias causes uncritical acceptance.^33^ Both failures undermine collaboration. Importantly, trust in HMCD varies across stages because the nature of the output that humans must rely upon changes. At attribute determination, trust concerns whether discovered features are genuinely relevant. At weight assignment, the question is whether data-driven weights reflect actual priorities rather than historical biases. At information aggregation, it is whether opaque fusion algorithms produce reliable assessments. At decision-making, trust concerns whether rankings align with human values and contextual realities. Explanation is the primary instrument for calibrating trust, with the required type shifting from justification of feature relevance at early stages to tracing of aggregation logic and ranking rationale at later stages.^6^
(4) Authority and responsibility migration: in traditional decision-making, authority over choices and accountability for outcomes reside in the same agent. HMCD can separate them. A machine may generate recommendations that effectively determine outcomes while a human nominally approves, leaving responsibility contested when errors occur.^34^ The Levels of Automation framework treats authority as a continuous spectrum from full human control to full machine autonomy,^35^ while team cognition research adds that effective collaboration requires shared mental models, clear boundaries, and traceable responsibility chains.^36^ The authority mechanism examines how decision power is distributed at each stage, when control transfers between agents, and how accountability should be attributed when outcomes are undesirable.

**Figure 2:**
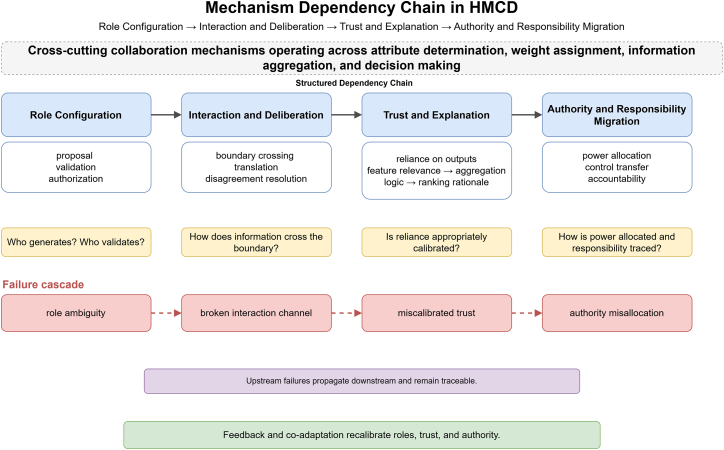
Mechanism dependency chain in HMCD The four collaboration mechanisms form a directed dependency chain rather than independent dimensions. Role configuration defines what information must cross the human-machine boundary, interaction and deliberation determine whether that information transfers with sufficient fidelity, trust and explanation calibrate human reliance on the resulting outputs, and authority and responsibility migration allocate decision power once trust is established. The upper row shows the forward enabling relations, the middle row formulates the diagnostic question each mechanism addresses, and the lower row traces the corresponding failure cascade through which role ambiguity propagates downstream into authority misallocation. Feedback and co-adaptation provide the recalibration path that keeps the chain operational.

These four mechanisms form a structured dependency chain rather than independent dimensions. Role configuration determines who generates and validates outputs, defining what information must cross the human-machine boundary. Interaction quality determines whether that information is transmitted with sufficient fidelity. Trust calibration depends on interaction quality, because humans cannot develop appropriate reliance on outputs they do not understand. Authority allocation presupposes calibrated trust, since rational delegation requires knowing when machine outputs are reliable. Failures therefore cascade in a predictable direction. If roles are ambiguously configured so that neither agent takes clear responsibility for attribute validation, the channel for flagging questionable features is never established, trust defaults to uncalibrated acceptance or rejection, and authority over attribute finalization becomes either too cautious or too permissive. Figure 2 illustrates this chain, showing both the forward linkages through which each mechanism enables the next and the failure cascade through which deficiencies propagate downstream.

#### Co-adaptation and framework integration

The four mechanisms described earlier are not static properties but evolve through repeated interaction. Co-adaptation theory describes how human users learn AI output characteristics and adjust their strategies, while AI systems refine recommendations based on interaction data.^37^^,^^38^ This mutual adjustment recalibrates role configurations, trust levels, and authority allocations over time.^39^ Feedback is the vehicle through which co-adaptation operates. Failed decisions may reveal omitted attributes, prompting expansion of the attribute set. Persistent errors may signal misaligned weights requiring recalibration. Without effective feedback, errors at early stages become embedded as permanent biases and the system loses adaptive capacity.

### Stage-specific collaboration analysis

**Figure 3:**
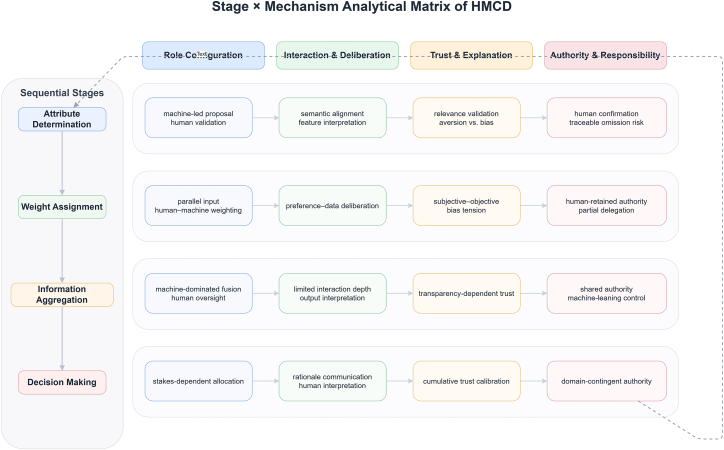
Stage × mechanism analytical matrix of HMCD The matrix maps the four collaboration mechanisms onto each of the four sequential stages, yielding sixteen cells that characterize stage-specific collaboration patterns. Each cell records the dominant configuration observed in the reviewed literature, including the typical division of proposal and validation roles, the prevailing interaction mode, the principal trust risk, and the locus of decision authority. Reading the matrix by row reveals how each mechanism evolves as the decision process advances, while reading by column shows which mechanisms are most consequential at a given stage. The matrix supports cross-stage comparison and guides method selection by linking design choices to the collaboration requirements of each stage.

This section examines how the four collaboration mechanisms identified in the “four cross-cutting collaboration mechanisms” section operate within each decision stage, analyzing stage-specific patterns of role configuration, interaction, trust calibration, and authority allocation. Figure 3 provides an overview of this analysis by mapping the four mechanisms onto each stage.

#### Attribute determination

Attribute determination specifies decision-relevant dimensions, criteria, or features, transforming complex problems into structured representations. The quality of this transformation constrains all subsequent stages.

Collaboration at this stage reconciles two modes of attribute discovery. Humans define high-level criteria based on domain expertise and value considerations, while machines extract latent features through data-driven pattern recognition. The central challenge is semantic alignment. Machine-discovered features typically lack interpretable labels, and determining whether statistical patterns correspond to meaningful decision factors requires structured exchange between agents. Context determines how this exchange is configured. In data-rich environments, machines lead discovery and humans serve as semantic filters. In knowledge-intensive domains, humans dominate attribute definition and machines assist with information retrieval. LLMs introduce a qualitatively new configuration by proposing decision dimensions in natural language, enabling conversational co-construction of attribute sets.^40^^,^^41^^,^^42^ However, this capability introduces hallucination risks, as models may generate plausible but fabricated attributes that enter the decision space without empirical grounding.^43^^,^^44^^,^^45^

Trust calibration must address two opposing failures: algorithm aversion that rejects counterintuitive but valid attributes^32^ and automation bias that accepts noise features uncritically.^33^ Final confirmation authority typically resides with humans because attribute definition involves value judgments, while accountability for omissions must remain traceable. Failures at this stage propagate silently, since an incomplete attribute set constrains all downstream analysis to irrelevant or distorted dimensions.

#### Weight assignment

Weight assignment determines the relative importance of identified attributes and, in group contexts, the relative influence of individual decision-makers.

The central tension at this stage is the conflict between subjective and objective weight sources. Humans express preferences through pairwise comparisons and value trade-off articulation that capture priorities data cannot encode. Machines perform consistency verification, derive objective weights from data distributions, and optimize weight vectors under logical constraints. When these two sources diverge, effective collaboration requires genuine deliberation rather than mechanical compromise. Machines can present contrastive analyses showing how different weight configurations alter final outcomes, enabling humans to make informed decisions about which preferences to maintain and which to revise. In group decision contexts, deliberation extends to negotiating preferences among multiple decision-makers with differing expertise and stakes. LLMs may reduce the cognitive burden of structured preference elicitation by serving as conversational negotiation interfaces, though the fidelity of preference capture through natural language remains an open question.

Trust risks manifest in two directions. Automation bias leads humans to accept data-driven weights that embed historical biases, while algorithm aversion causes rejection of objective weights even when algorithmic estimation is demonstrably more accurate. Because weights encode value judgments, humans should retain final authority. Yet in large-scale problems, partial delegation to machines becomes necessary, making traceability of weight origins essential for procedural legitimacy. Failures in weight assignment propagate directly into information aggregation, systematically skewing evaluative integration and final rankings.

#### Information aggregation

Information aggregation integrates evaluative inputs from human and machine agents into unified assessments, synthesizing qualitative judgments, quantitative estimates, and uncertain evaluations into coherent preference structures.

This stage is computationally dominated by machine intelligence. The human role shifts from active input provider to supervisor and validator, contributing through output interpretation, assumption checking, and contextual judgment. The primary collaboration risk is that this shift degenerates into passive acceptance of opaque machine outputs. Complex aggregation models often generate accurate predictions while offering limited insight into their reasoning. When humans cannot understand how aggregation results derive from individual inputs, collaboration polarizes into uncritical acceptance or wholesale rejection. Explanation methods that trace how fusion results emerge from input weights and evidence combinations are therefore essential for maintaining meaningful oversight. Upstream errors embedded through attribute omission or weight misalignment become progressively harder to detect at this stage, because the mathematical operations that produce aggregated outputs obscure the origins of upstream biases.

#### Decision-making

Decision-making encompasses the final selection among alternatives, implementation of chosen actions, and monitoring of outcomes for feedback integration.

Machine agents execute ranking computations while human decision-makers select methods, specify parameters, interpret results, and authorize final choices. The balance between these roles depends on domain stakes and accountability requirements rather than technical capability alone. In high-stakes contexts such as clinical treatment and military operations, humans must retain ultimate authority because accountability for irreversible consequences cannot be delegated. In routine contexts such as quality assessment, machines may assume greater autonomy under supervisory oversight. Effective collaboration requires that machine recommendations convey not only rankings but also the rationale linking outcomes to upstream weights and evidence. When multiple alternatives yield comparable scores, the system should present options transparently for human deliberation rather than resolving ambiguity autonomously. Trust at this stage has a cumulative character, reflecting calibration built across all preceding stages.

This stage also represents the primary entry point for feedback into the entire HMCD process. When outcomes deviate from expectations, the four-stage decomposition provides a structured basis for diagnosing whether the failure originated in attribute omission, weight misalignment, aggregation distortion, or execution error. In high-velocity environments such as real-time combat or emergency medical response, stages compress into extremely short time windows, requiring that collaboration mechanisms be pre-established through system design rather than negotiated during each decision episode.

## Methods and applications analyzed through the framework

The four stages do not operate in isolation. Upstream decisions cumulatively constrain downstream operations, while feedback enables downstream outcomes to trigger upstream corrections, and the four mechanisms evolve through repeated interaction.

### Attribute determination

#### Deep learning

Deep learning extracts latent features from complex data through multi-layer representation learning, in which neural networks progressively transform raw inputs into higher level abstractions by adjusting connection weights through iterative training.^46^^,^^47^^,^^48^ This capacity makes deep learning a primary machine-side tool for attribute determination in HMCD.

The core collaboration challenge is semantic alignment. Neural networks extract features that lack interpretable labels, and post hoc tools such as Shapley additive explanations (SHAP)^49^ or local interpretable model-agnostic explanations (LIME)^50^ can only partially bridge this gap by indicating which features matter statistically without confirming whether they correspond to meaningful decision dimensions. Trust miscalibration manifests as algorithm aversion when humans reject counterintuitive but valid attributes or as automation bias when noise features are accepted uncritically. Humans operationalize the gating function through visualization tools such as saliency or attention maps and through small held-out validation sets, which give experts a tractable inspection target without confirming semantic relevance.

The three main paradigms configure collaboration differently.^51^ Supervised learning gives humans upstream authority through data labeling, but label quality constrains attribute validity. Unsupervised learning shifts discovery to machines with humans validating downstream, though the absence of ground truth makes validation harder. Deep reinforcement learning distributes control through reward design, but misspecified rewards produce misleading feature representations. Compromised attributes then propagate into weight assignment and amplify during aggregation.

#### Feature selection

Feature selection identifies relevant attributes from predefined feature spaces, reducing dimensionality and improving interpretability.^52^^,^^53^ Filter methods evaluate features through statistical criteria independent of learning algorithms. Wrapper methods assess subsets based on predictive model performance. Embedded methods integrate selection within model training through regularization or importance weighting.^54^^,^^55^

Feature selection presents a more structured collaboration pattern than deep learning because the candidate space is human defined. Machines narrow it using statistical criteria while humans make definitive selections based on domain knowledge. Trust calibration benefits from transparent quantifiable criteria, though this transparency may generate overreliance on statistics without questioning whether the data adequately represent the decision context. Filter methods involve single-pass evaluation with limited negotiation, while wrapper and embedded methods enable progressive alignment through model performance feedback. Excluded attributes are permanently absent from downstream stages, making selection errors difficult to detect later.

#### Knowledge graphs

Knowledge graphs represent domain knowledge as networks of entities and relationships, transforming fragmented information into structured, queryable representations that serve as shared cognitive artifacts between human and machine agents.^56^^,^^57^^,^^58^^,^^59^

Knowledge graphs support bidirectional collaboration. Humans design ontologies while machines perform entity extraction and relation inference.^60^ The entity-relation structure supports trust calibration because humans can inspect machine-inferred relationships directly, reducing the semantic alignment problems that affect deep learning. Authority over knowledge structure is predominantly human-held through ontology design, though machines increasingly contribute through automated completion. Integrating LLMs with knowledge graphs creates a new paradigm in which LLMs accelerate entity and relation generation while the graph provides structured grounding that constrains outputs and mitigates hallucination,^61^^,^^62^ although LLM-generated elements require ongoing human validation.

Table 1 reveals a transparency gradient that determines feasible collaboration depth. Semantic alignment difficulty rises from knowledge graphs through feature selection to deep learning, and the interaction pattern follows the same gradient, from co-construction to sequential handoff to post hoc review. Method selection should therefore consider both data characteristics and the collaboration capacity available in a given context. Persistent downstream anomalies in weight assignment or aggregation can also signal attribute redundancy or omission, partially compensating for failures at this stage.

**Table 1:** Comparison of attribute determination methods in HMCD

Dimension	Deep learning	Feature selection	Knowledge graphs
Core mechanism	latent attribute discovery through multi-layer representation learning	statistical screening and ranking of candidates from a predefined feature space	semantic structuring of entities and relational dependencies
Collaboration pattern	machine-initiated, human-gated	human-scoped, machine-refined	co-constructive, iterative
Semantic alignment risk	high; latent features lack semantic correspondence to human decision concepts	low to moderate; human-defined space preserves semantics, but statistical and domain relevance may diverge	low; explicit entity-relation structures ensure transparency; risk shifts to completeness
Conditions and constraints	large-scale data needed; human validators require interpretive expertise; degrades when features resist interpretation	partially known attribute sets needed; effective when domain knowledge provides reasonable initial space	established vocabularies needed; resource intensive; most valuable with strong relational dependencies

### Weight assignment

#### Analytic hierarchy process

The analytic hierarchy process (AHP) structures weight assignment through iterative human-machine collaboration.^63^ Humans provide pairwise comparison matrices reflecting value priorities, while machines calculate eigenvectors, derive weights, and verify consistency ratios. This creates a distinctive feedback loop in which machines flag consistency violations and humans revise judgments, enabling progressive alignment between expressed preferences and logical coherence. Trust calibration benefits from procedural transparency, since weight derivation is traceable at every step, but this transparency may foster overconfidence in subjective preferences that carry systematic biases such as anchoring. Authority is firmly human-held, with machines performing computational and verification functions. AHP assumes static criteria and stable preferences, so when contexts shift rapidly, the comparison matrices become outdated and the method offers no incremental recalibration without repeating the elicitation process. Biases embedded in comparison matrices propagate directly into aggregation.

#### Entropy weight method

The entropy weight method (EWM) derives attribute importance from data variability, assigning higher weights to attributes that carry more discriminative information.^64^ Machines perform entropy calculation and weight derivation autonomously while humans validate alignment with domain priorities.^65^ Analytical initiative concentrates almost entirely on the machine side, and interaction is largely one-directional. Humans receive completed weight vectors rather than participating in their construction, which restricts mid-process deliberation. Objectivity supports reproducibility but carries an implicit assumption that data variability equals decision importance, so when this assumption is invalid, the distortion amplifies during aggregation without clear attribution. Authority is formally shared, but in practice, machine-generated weights serve as defaults that humans accept without substantive revision, creating a subtle authority migration in which machines effectively determine weight structures while humans retain nominal approval.

#### Combination weighting method

The combination weighting method integrates subjective and objective approaches through linear combination or optimization-based synthesis.^66^ By making the tension between value-based and data-driven weights an explicit object of negotiation, this method addresses a limitation shared by both AHP and the entropy method. Humans provide preference-based weights, while machines compute objective weights and optimize integration parameters. Machines can present comparative analyses showing how different weighting schemes affect final outcomes, enabling informed human trade-offs. This dual-source structure enables balanced trust calibration, as neither weight source dominates unchecked. However, the integration ratio itself constitutes a meta-decision that is often set algorithmically without explicit justification, potentially introducing an unexamined source of bias.

Table 2 shows that the three methods configure the value-data tension differently and each carries a characteristic trust vulnerability. AHP may mask preference bias behind procedural transparency, EWM may generate misplaced confidence in statistical variability, and the combination method introduces opacity at the integration point. Downstream feedback can signal weight misalignment but only when sensitivity analysis makes the link between weight configurations and decision quality explicit.

**Table 2:** Comparison of weight assignment methods in HMCD

Dimension	AHP	EWM	Combination weighting method
Core mechanism	subjective preference elicitation and structured weight derivation	objective weight derivation based on data variability	integration of subjective and objective weights
Collaboration pattern	human-driven, machine-verified	machine-initiated, human-supervised	human approves; parallel-input, machine-mediated
Value-data tension	fully subjective; no data correction; systematic expert biases undetectable within the method	fully objective; ignores value priorities; may overweight variable but decision-irrelevant attributes	partially resolved, but integration ratio becomes a meta-decision often set algorithmically without justification
Conditions and constraints	manageable attribute count; auditable weight justification required	sufficient quantitative data with meaningful variability; stakeholders accept data-driven logic	both domain expertise and quantitative data available; context-adaptive integration ratio needed

### Information aggregation

#### Probabilistic methods

Probabilistic methods model uncertainty through frameworks such as Markov decision processes,^67^ Gaussian processes,^68^ Bayesian optimization,^69^ and belief function theory.^70^ The collaboration follows a prior-posterior pattern in which humans encode initial beliefs and validate distributional assumptions while machines refine estimates through data-driven updating.

The role configuration at this stage assigns humans a framing function and machines an execution function. Humans shape the direction of inference by specifying priors, selecting model structures, and setting distributional assumptions. Machines perform updating, computation, and uncertainty propagation within these human-defined boundaries. This structure preserves human influence on inferential direction while delegating computational complexity. However, the interaction between agents is limited in depth. Once priors and assumptions are specified, the machine-driven updating process typically proceeds without further human input until outputs are delivered, leaving few opportunities for mid-process deliberation or adjustment. Trust depends on assumption transparency. When distributional assumptions diverge from real conditions, humans may lack the information needed to judge output reliability. Authority is shared, with humans shaping inferential direction and machines determining update magnitude. Misspecified assumptions propagate into decision-making as apparently principled but misleading recommendations.

#### Ensemble learning

Ensemble learning aggregates predictions from multiple models through techniques such as bagging, stacking, and boosting to improve robustness and accuracy.^71^^,^^72^ Machines automatically integrate diverse model outputs without explicit human intervention during aggregation. Human participation is largely post hoc, consisting of base model selection, output interpretation, and domain validation.

This method illustrates the challenge of maintaining meaningful human oversight when machines dominate computation. The aggregation logic may obscure how individual predictions contribute to final outputs, creating a pronounced interpretability gap. Humans may either accept opaque outputs uncritically or reject them entirely, unable to distinguish genuine patterns from model artifacts. Authority over fusion parameters is predominantly machine-held, with humans retaining oversight and veto rights but lacking practical means to intervene during the aggregation process. Errors in upstream attribute selection or weight assignment are absorbed into ensemble inputs and may be masked by the apparent robustness of combined outputs.^73^

#### Evidence reasoning

Evidence reasoning, rooted in Dempster-Shafer theory, assigns belief masses to subsets of possible outcomes^74^ rather than requiring precise probability assignments for individual elements.^75^ This allows explicit representation of both partial knowledge and ignorance, providing a more flexible framework for combining uncertain and incomplete information from multiple sources.

This approach enables a more natural form of human-machine interaction than either probabilistic or ensemble methods. Humans can express partial beliefs without providing precise probability distributions, and machines handle evidence combination and conflict resolution computationally. Trust calibration is well supported because humans can directly inspect which evidence sources contribute to aggregated beliefs and where conflicts arise, enabling meaningful oversight even when computational combination is complex. The explicit representation of ignorance and conflict provides downstream decision-making with richer uncertainty information than point estimates from other aggregation methods.^76^

Table 3 shows a transparency gradient. Evidence reasoning supports genuine collaboration through inspectable belief assignments, probabilistic methods offer moderate transparency through defined mathematical semantics, and ensemble learning offers the least because individual model contributions resist decomposition. The choice of method therefore determines not only computational outcomes but also the feasible mode of human participation. Anomalous outputs should trigger re-examination of upstream inputs, since they may signal attribute omission or weight misalignment.

**Table 3:** Comparison of information aggregation methods in HMCD

Dimension	Probabilistic methods	Ensemble learning	Evidence reasoning
Core mechanism	uncertainty-driven fusion through probabilistic modeling and belief updating	multi-model output aggregation for robustness	belief-function-based integration of heterogeneous and conflicting evidence
Collaboration pattern	iterative belief calibration	machine-dominated, human-bounded	dual-input, conflict-mediated
Transparency and interpretability	moderate; defined mathematical semantics, but complex hierarchical models can become opaque	low; individual model contributions to final output are opaque; post hoc explanations miss aggregation logic	high; belief assignments, conflict levels, and results are explicitly quantifiable and traceable
Conditions and constraints	quantifiable uncertainty needed; human experts must specify meaningful priors	data-rich environments needed; requires post hoc explanation for human oversight	incomplete or multi-source information; human experts must specify belief boundaries

### Decision-making

#### Game theory

Game theory formalizes strategic interactions between human and machine agents in both cooperative and competitive decision scenarios.^77^ Humans define payoff structures reflecting organizational values and risk preferences while machines compute equilibria and simulate scenarios.

The role configuration is distinctively iterative. Rather than a one-directional handoff, both agents progressively refine the problem representation. Humans specify payoff structures and strategic constraints, machines identify equilibrium solutions, and humans evaluate whether these solutions align with intended objectives. When they do not, the payoff structure is revised and the cycle repeats. This iterative refinement constitutes a form of deliberation that is absent from most other decision-making methods. Trust calibration is supported by the formal transparency of equilibrium analysis. However, solution sensitivity to payoff assumptions means that small misspecifications in human inputs can produce substantially different machine-computed outcomes. This sensitivity demands careful attention to whether the problem framing genuinely captures decision realities. Authority over problem framing and final choice selection remains with humans while machines handle computational exploration.

#### Optimization methods

Optimization methods identify satisfactory solutions from complex decision spaces when exhaustive search is infeasible. Metaheuristic algorithms such as genetic algorithms and swarm-based methods provide flexible search guidance under incomplete information.^78^ Humans define objective functions and constraints while machines explore the solution space within those boundaries.

The role configuration follows a clear partition. Humans hold definitional authority over goals and constraints, machines hold search authority within those boundaries, and humans retain approval authority over proposed solutions. The interaction is limited in depth, as humans typically cannot observe or steer the search process in real time. Trust calibration depends on whether humans can assess solution quality without provable optimality guarantees, a judgment that requires domain expertise rather than algorithmic understanding. A significant limitation is that optimization methods assume stable objective functions. When decision goals shift during the search process, as frequently occurs in dynamic operational environments, the method provides no built-in mechanism for real-time goal adaptation. This rigidity constrains their applicability in time-sensitive HMCD contexts.

#### Multi-criteria decision methods

MCDM methods integrate attribute weights and evaluation information to rank alternatives through approaches such as technique for order preference by similarity to ideal solution (TOPSIS), multi-criteria optimization and compromise solution (VIKOR), elimination and choice translating reality (ELECTRE), and preference ranking organization method for enrichment evaluation (PROMETHEE).^63^^,^^79^^,^^80^ Each method embeds different assumptions about how trade-offs should be resolved, and selecting among them constitutes a value judgment that machines cannot make independently.

The interaction centers on method and parameter choices that encode human priorities. Selecting VIKOR over TOPSIS, or adjusting compromise coefficients, reflects preferences about how trade-offs should be handled. Machines perform ranking calculations while humans select methods, specify parameters, interpret results, and authorize final selections. MCDM methods offer high transparency and reproducibility, supporting trust through traceable ranking logic. However, these methods assume static criteria and stable preference structures, which limit their applicability when decision contexts evolve rapidly. TOPSIS and VIKOR, for example, rely on fixed ideal and anti-ideal reference points that may become invalid as conditions change yet provide no mechanism for dynamic recalibration during the decision process. Their effectiveness also depends entirely on upstream input quality from attribute determination, weight assignment, and information aggregation, making them the point where upstream errors become most visible.

Table 4 shows that the three methods partition human-machine authority differently and therefore differ in accountability traceability. Game theory distributes authority through iterative problem formulation, which makes failure attribution difficult because errors may originate in payoff specification, equilibrium computation, or selection among multiple equilibria. Optimization methods partition authority cleanly between human goal specification and machine search, providing a clearer diagnostic structure when failures occur. MCDM methods maintain human dominance throughout, and their sequential logic makes it easiest to trace whether failures lie in method selection, parameter specification, or upstream input quality.

**Table 4:** Comparison of decision-making methods in HMCD

Dimension	Game theory	Optimization methods	MCDM
Core mechanism	strategic decision-making under multi-agent interaction	constrained solution search through metaheuristic or mathematical programming	multi-attribute ranking and selection
Collaboration pattern	human-framed, machine-computed	human-specified, machine-executed	human-dominant throughout
Authority allocation	human-anchored; machine computes options, human retains final strategic commitment	machine holds search authority, human holds definitional and approval authority	machine computes rankings, human retains interpretive and selection authority
Conditions and constraints	specifiable payoff structures; less suitable for unquantifiable values	clearly definable objectives and constraints; best for operational decisions with precise goals	well-structured upstream inputs required; transparent logic; less effective under rapid change

### Domain applications

#### Management science

In management science, HMCD is predominantly human dominated. Humans retain decision authority over goal setting, value judgment, and final approval, while AI systems support analysis, prediction, and evaluation.^81^^,^^82^

The most distinctive challenges arise at weight assignment and decision-making. Weight assignment requires negotiation between managerial preferences rooted in experience and objective weights derived from data distributions, a process complicated by bounded rationality and by the difficulty of articulating implicit value trade-offs in quantifiable form.^83^ Unlike domains with well-defined performance metrics, management decisions often involve incommensurable objectives, making subjective weighting unavoidable yet difficult to validate. At the decision stage, authority is formally shared but governance risks are most pronounced, since unclear allocation of accountability between managers and AI recommendations raises organizational control concerns when algorithmic suggestions conflict with managerial intuition. When AI outputs lack transparency, managers either defer uncritically or dismiss recommendations in favor of familiar heuristics, undermining complementarity in both cases. Attribute determination and information aggregation follow more conventional patterns, with managers defining criteria and machines handling analytical integration. Feedback cycles are slow due to long strategic horizons and organizational inertia.

#### Military

Military HMCD operates under hierarchical command structures where human commanders retain control over mission objectives, rules of engagement, and execution decisions. Intelligent systems support situational assessment, option generation, and resource optimization.^84^

The most acute challenges arise at information aggregation and decision-making, both under severe time pressure. Multi-source intelligence fusion operates under the fog of war where source reliability is uncertain. Commander trust in fused outputs directly governs decision quality, and misplaced trust can lead to targeting errors with irreversible consequences. The trust problem differs from civilian domains because adversaries may deliberately manipulate the information environment to exploit automation bias.^85^^,^^86^^,^^87^ In decision-making, whether autonomous weapon systems may act independently when communications are severed represents the most acute authority migration challenge in any HMCD domain,^88^ with ambiguous boundaries between recommendation and autonomous action creating accountability gaps that current doctrinal and technical frameworks do not resolve. Sensor data may also introduce new attributes autonomously, creating a conflict between human-defined and machine-discovered variables, and the trade-off between humanitarian considerations and military efficiency requires deliberation mechanisms that combat pressure severely constrains. Feedback is high latency and subject to adversarial interference, so military HMCD must perform reliably without extended iterative learning, making pre-established collaboration protocols essential.

#### Manufacturing

In manufacturing HMCD, decision authority is typically distributed. Humans retain control over production goals, quality standards, and exception handling, while machines dominate real-time data processing, condition monitoring, and local optimization.^89^^,^^90^ Recent developments in embodied intelligence extend this paradigm by integrating perception, decision, and physical action within robotic systems, deepening human-machine coupling in production settings.^91^^,^^92^^,^^93^^,^^94^

The most distinctive feature is the tight coupling between information aggregation and execution as high-speed collaboration stages. Machine agents dominate through continuous data fusion and embodied feedback, while human oversight is supervisory. Errors in sensing, model drift, or misaligned embodied actions may propagate rapidly across production chains, amplifying local deviations into system-level failures. Embodied intelligence intensifies this dynamic by shortening the perception-action loop and increasing local autonomy, which accelerates both the benefits and risks of machine-dominated aggregation. Interpretability of adaptive embodied behaviors remains limited, hindering human ability to diagnose and intervene when actions deviate from intended parameters. Attribute determination and weight assignment are often pre-structured through system design, embedding implicit preferences that resist detection and revision. Feedback is continuous and embedded in monitoring loops, providing more rapid co-adaptation than in other domains.^95^

#### Medicine

In medical HMCD, decision authority remains clinician centered. Physicians retain responsibility for defining clinically meaningful variables, exercising value judgments, and authorizing final treatment decisions, while AI systems support information aggregation, pattern recognition, and outcome prediction.^96^^,^^97^

The most critical challenges concentrate at information aggregation and the trust mechanism that mediates its use. Machine agents integrate heterogeneous clinical data and generate probabilistic assessments, but model opacity complicates validation, accountability, and clinical communication.^98^ Physicians require not only accurate outputs but also understandable rationales to justify decisions to patients and colleagues. Trust calibration operates under high stakes, since overreliance may lead to missed diagnoses while excessive skepticism may discard valid insights. Medical HMCD also faces regulatory and ethical constraints on feedback and co-adaptation that do not apply in other domains. Clinical outcome feedback is often delayed, incomplete, and constrained by privacy requirements.^99^^,^^100^ Adaptive learning from patient data raises concerns about data security and informed consent that are absent from manufacturing or management contexts. Attribute determination and weight assignment are strongly clinician dominated, with value judgments about risk, benefit, and patient preferences taking precedence over purely data-driven weighting.

#### Cross-domain comparative analysis

Table 5 summarizes these findings along five dimensions. Three cross-cutting patterns emerge. Information aggregation is consistently the stage of highest machine autonomy, but the sources of trust vulnerability differ across domains, from adversarial manipulation in the military to model opacity in medicine and sensor drift in manufacturing. Authority migration sensitivity follows a gradient that tracks consequence reversibility, with military and medical domains requiring the highest safeguards. Feedback loop speed varies by orders of magnitude, from second-level sensor loops in manufacturing to years-long strategic evaluation in management. The stage-mechanism structure therefore applies across domains but requires domain-specific calibration.

**Table 5:** Cross-domain comparison of HMCD collaboration patterns

Dimension	Management science	Military	Manufacturing	Medicine
Dominant role configuration	human dominated across most stages; machine supports analysis and prediction	hierarchical human command with machine support for situational assessment and option generation	distributed; machine dominates real-time operations while humans control goals and exceptions	strongly human dominated with selective machine augmentation in information processing
Primary trust vulnerability	decision-making, where organizational accountability demands constrain reliance on algorithmic recommendations	information aggregation, where fog of war undermines confidence in fused intelligence	information aggregation and execution, where sensor uncertainty and model drift reduce output reliability	information aggregation, where model opacity limits clinical acceptance
Authority migration sensitivity	low to moderate; governance structures permit gradual and reversible delegation	very high; misallocation may cause irreversible operational consequences	moderate; process coupling amplifies local errors but outcomes are typically recoverable	high; patient safety and legal liability require sustained clinician control
Feedback loop characteristics	slow; long strategic horizons and organizational inertia delay outcome evaluation	high latency with asymmetric availability; adversarial interference disrupts real-time feedback	fast and embedded; sensor-level loops enable second-level adaptation	delayed and constrained by regulatory, ethical, and privacy requirements
Co-adaptation trajectory	gradual; trust and roles evolve incrementally with organizational experience	constrained; reliable performance required without extended iterative learning	continuous and embedded through production monitoring and operator-machine adjustment	cautious; clinical validation requirements and institutional conservatism slow mutual adaptation

## Discussion

### Core challenges

Despite the proliferation of HMCD methods and applications, several fundamental challenges constrain practical efficacy and broader adoption. These challenges span technological, human, and institutional dimensions, and each manifests with varying intensity across the four collaboration mechanisms and decision stages identified in the framework.

#### Technical challenges

The trade-off between interpretability and performance most acutely affects the trust mechanism during information aggregation. As model complexity grows, interpretability declines, making it difficult for human decision-makers to understand, validate, and contest algorithmic recommendations.^101^ This is particularly consequential at the information aggregation stage, where machines dominate computation and humans risk becoming passive recipients of opaque outputs. When aggregation logic remains invisible, trust polarizes into uncritical acceptance or wholesale rejection.

Algorithmic bias propagates through the role configuration mechanism at foundational stages. In attribute determination, machine-discovered features may reflect historical patterns that encode discriminatory dimensions,^102^^,^^103^ shaping the decision space in ways humans cannot easily detect. In weight assignment, data-driven importance scores may perpetuate past biases. Because these biases are introduced early, they amplify through subsequent stages and become increasingly difficult to attribute to their source. Structured approaches combining algorithmic auditing, diverse training data, and continuous monitoring across stages are needed.

Limited adaptability in dynamic environments reflects deficiencies in the feedback mechanism. Traditional methods such as AHP and TOPSIS assume static criteria and stable preferences, while current AI systems often lack mechanisms for incremental learning and real-time recalibration. This gap indicates that most HMCD systems remain predominantly feedforward, with weak cross-stage feedback loops that prevent timely adjustment as conditions evolve. Reinforcement learning, causal inference, and adaptive control strategies offer potential paths toward more responsive configurations.^104^^,^^105^

LLMs present a distinct challenge that cuts across the preceding three issues by blurring traditional capability boundaries between humans and machines. They are neither rule-based machines nor accountable human agents but function as collaborative intermediaries with linguistic capabilities, and this position reshapes each of the four mechanisms.^106^ In role configuration, they enter the decision process as a third type of agent that can simultaneously propose candidate outputs and articulate validation rationales. In interaction and deliberation, they translate between technical outputs and natural language reasoning, which lowers communication costs but makes fluent errors harder to detect. In trust and explanation, the rationales they generate must be checked against underlying data and model traces rather than judged by surface plausibility. In authority and responsibility migration, they blur authorship when recommendations appear jointly produced by human prompts and machine responses, leaving accountability difficult to attribute when errors occur.

These cross-mechanism effects manifest as concrete failure modes at specific stages. Hallucination at attribute determination may introduce fabricated attributes that propagate undetected, while sycophancy at weight assignment reinforces rather than challenges human preferences^107^ Both failures appear as linguistically fluent outputs that humans find difficult to separate from valid contributions, so safeguards must be designed for the stage at which the failure originates rather than added uniformly. Source grounding constrains attribute proposals to evidence that humans can trace, independence checks during preference elicitation guard against weight estimates that merely echo prior human inputs, and evidence-linked explanations during aggregation and final choice expose the chain from raw inputs to recommended outcomes. Together, these safeguards keep LLM-mediated collaboration within the dependency chain described by the four mechanisms rather than allowing it to bypass the chain.

#### Human factors challenges

Trust calibration difficulties pervade all stages, but the consequences are most severe at information aggregation and decision-making, where miscalibrated trust meets high-stakes outcomes. The design challenge is not eliminating miscalibration but providing stage-appropriate calibration. Aggregation requires explanation methods that trace fusion logic, while decision-making requires transparent presentation of ranking rationale and uncertainty.

Cognitive load mainly affects the interaction mechanism during attribute determination and information aggregation. When information presentation exceeds human cognitive capacities, decision-makers experience overload and produce delayed or suboptimal decisions.^108^ This is most pronounced when humans evaluate machine-generated attribute candidates or interpret complex aggregation outputs under time pressure. Interaction designs should preserve the asymmetry between human contextual awareness and machine computational capacity rather than make both agents process the same information.

#### Institutional challenges

Ambiguity in responsibility attribution engages the authority mechanism directly. When decisions emerge from human-machine interaction, errors may originate at attribute determination, propagate through subsequent stages, and manifest only at decision-making, which makes responsibility allocation unclear. The four-stage framework with documented role configurations supports retrospective responsibility analysis.

Existing regulations address ethical and legal constraints unevenly. Fairness requirements constrain role configuration and weight assignment, transparency mandates shape trust and explanation, and privacy protections limit interaction. The absence of clear institutional guidelines discourages deployment in safety-critical domains.

### Failure modes across the four stages of HMCD

The mechanism dependency chain introduced in the “four cross-cutting collaboration mechanisms” section implies that failures rarely remain contained within a single stage. The sequential structure creates natural transmission pathways for both forward and backward error propagation, and weak feedback allows early errors to embed as permanent biases.

**Figure 4:**
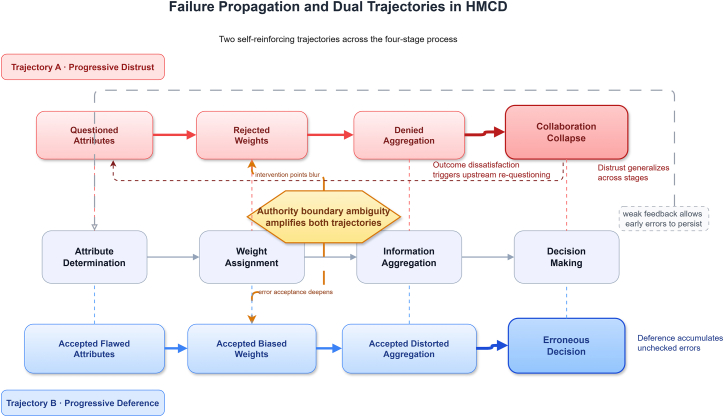
Failure propagation and dual trajectories in HMCD Failures in HMCD seldom remain confined to a single stage and follow two self-reinforcing trajectories. Trajectory A, progressive distrust, is backward looking. An unsatisfactory final decision triggers retrospective questioning of aggregation, weights, and attributes, which generalizes into broader rejection of the system. Trajectory B, progressive deference, is forward moving. Uncorrected acceptance of flawed attributes leads to biased weights, distorted aggregation, and ultimately an erroneous decision. Authority boundary ambiguity amplifies both trajectories because no agent holds clear responsibility for reopening earlier stages or interrupting unchecked propagation. Weak feedback allows early errors to embed as permanent biases, which is why proactive design intervention at upstream stages is essential.

Trust failures follow two causal paths. Progressive distrust is backward looking. A poor or unexplained final decision creates negative feedback, prompting users to reassess whether the attributes were relevant, whether weights reflected real priorities, and whether aggregation distorted evidence. Progressive deference is forward moving. Early machine outputs are accepted without validation, so flawed attributes shape biased weights, biased weights distort aggregation, and distorted aggregation reaches decision-making as if it were reliable. Authority ambiguity strengthens both paths because no agent has a clear duty to reopen earlier stages or interrupt unchecked propagation. Figure 4 illustrates these two trajectories, with distrust shown as feedback from outcomes to upstream assumptions and deference shown as feedforward accumulation of uncorrected error.

### Future research directions

#### Stage-anchored research questions

The dual-layer framework provides a systematic basis for identifying research gaps by examining stage-mechanism intersections. The following questions represent high-priority openings exposed by this analysis.

Attribute determination needs interactive tools that support human-machine co-creation. Current systems present machine-discovered features as fixed outputs for review, but effective collaboration would let humans guide feature discovery while machines expand the search space. LLMs provide a natural-language interface for such co-creation, though hallucination risks require verification protocols that keep humans in control of the final attribute set.

Weight assignment needs algorithms that surface preference conflicts between subjective and objective weights and support structured negotiation. Value alignment techniques, particularly reinforcement learning from human feedback (RLHF), can encode human preferences into machine weight recommendations, but their application to multi-criteria contexts with competing stakeholder preferences remains underdeveloped. This is a direct connection between the alignment literature and HMCD research.

Information aggregation needs explanation methods tailored to fusion processes. Generic approaches do not adequately serve aggregation, where human oversight is most constrained and machine autonomy is highest. Methods that trace how aggregated outputs derive from input weights and evidence combinations would let humans validate fusion logic rather than accept or reject outputs as black boxes.

Decision-making needs principles for dynamic authority allocation that adjust across stages based on task characteristics, observed reliability, and cumulative trust. Current systems assign fixed authority levels, but effective collaboration may require authority to shift with performance and context. Automated outcome evaluation methods that direct feedback to the appropriate upstream stage are equally important, since current co-adaptation mechanisms receive limited empirical investigation.

#### Domain-specific research priorities

Building on the domain analyses in the “domain applications” section, this section identifies domain-specific research priorities that complement the cross-cutting questions in the “stage-anchored research questions” section.

In management science, the open problem is group decision-making in hybrid teams of human managers and AI agents. Current models assume homogeneous decision-makers, but hybrid teams need mechanisms for negotiating preferences across agents with fundamentally different cognitive and representational systems,^109^^,^^110^ particularly at the weight assignment stage where value trade-offs resist algorithmic resolution.

In military contexts, the priority is HMCD models resilient to adversarial manipulation. Military information environments may be deliberately distorted to exploit automation bias.^111^^,^^112^^,^^113^ Developing trust calibration methods that account for adversarial interference during information aggregation, rather than assuming benign data conditions, addresses a gap that civilian-origin HMCD approaches cannot fill.

In manufacturing, embodied intelligent agents create new research problems. As collaborative robots become physical actors sharing workspace with operators, role configuration and authority allocation must accommodate real-time physical interaction rather than only informational exchange. Interpretable embodied decision processes that let operators understand and intervene in adaptive robotic behaviors are essential for sustaining effective oversight.^114^^,^^115^

In medicine, the convergence of LLMs with clinical decision support creates both opportunity and risk. LLMs can serve as conversational interfaces for diagnostic querying, but alignment with clinical values requires domain-specific validation. Evaluation frameworks that assess not only predictive accuracy but also the fidelity of LLM-mediated explanations to underlying clinical evidence are an urgent priority given the pace of deployment.^116^^,^^117^

## Conclusion

This study presents a comprehensive review of HMCD methods and applications, organized around a dual-layer analytical framework that combines sequential stage decomposition with cross-cutting collaboration mechanisms. Using this framework, we synthesize methods and applications across management science, the military, healthcare, and manufacturing and identify where collaboration succeeds and where it breaks down.

Three principal findings emerge from this analysis. First, decision authority should vary systematically across stages rather than remain fixed. Human authority should be strongest at attribute determination, weight assignment, and high-stakes decision-making, where value judgments and accountability dominate. Machine autonomy is most appropriate at information aggregation and routine operational decisions. Authority at any stage must be supported by explanation for trust calibration and clear responsibility boundaries for accountability.

Second, collaboration failures originate from mechanism-level deficiencies rather than technical inadequacies and propagate across stages through mechanism dependencies. The two trajectories identified, progressive distrust leading to collaboration collapse and progressive deference leading to unchecked error accumulation, are both self-reinforcing and require proactive design intervention at early stages.

Third, no single decision-making method is universally optimal. Classical MCDM approaches offer transparency but assume static conditions, while learning-based methods provide adaptability at the cost of explainability. Effective HMCD systems require deliberate integration of methods matched to the collaboration requirements of each stage.

This review has several limitations. As a comprehensive narrative review rather than a systematic synthesis, the selection of studies emphasized conceptual representativeness over exhaustive coverage. The search was further restricted to Web of Science and four application domains, so relevant work in education, transportation, and agriculture may offer additional insights. The framework has not been empirically validated through controlled experiments, and its applicability to real-time systems with compressed stage transitions remains to be tested. Future research should prioritize empirical validation of the stage-mechanism structure and domain-calibrated implementations that translate the framework into operational design.

## Acknowledgments

This research was funded by 10.13039/501100012456National Social Science Fund of China (2025-SKJJ-B-047 and 2025-SKJJ-D-065).

## Declaration of interests

The authors declare no competing interests.
